# Cyclic Nucleotide-Specific Optogenetics Highlights Compartmentalization of the Sperm Flagellum into cAMP Microdomains

**DOI:** 10.3390/cells8070648

**Published:** 2019-06-27

**Authors:** Diana N. Raju, Jan N. Hansen, Sebastian Rassmann, Birthe Stüven, Jan F. Jikeli, Timo Strünker, Heinz G. Körschen, Andreas Möglich, Dagmar Wachten

**Affiliations:** 1 Institute of Innate Immunity, Biophysical Imaging, University Hospital Bonn, University of Bonn, 53127 Bonn, Germany; 2 Centrum für Reproduktionsmedizin und Andrologie (CeRA), Universitätsklinikum Münster, Universität Münster, 48129 Münster, Germany; 3 Lehrstuhl für Biochemie, Universität Bayreuth, 95447 Bayreuth, Germany; 4 Center of Advanced European Studies and Research (caesar), Molecular Sensory Systems, 53175 Bonn, Germany; 5 Research Center for Bio-Macromolecules, Universität Bayreuth, 95447 Bayreuth, Germany; 6 Bayreuth Center for Biochemistry & Molecular Biology, Universität Bayreuth, 95447 Bayreuth, Germany; 7 Center of Advanced European Studies and Research (caesar), Molecular Physiology, 53175 Bonn, Germany

**Keywords:** cilia, flagella, optogenetics, cAMP, beat pattern, motility, navigation, sperm

## Abstract

Inside the female genital tract, mammalian sperm undergo a maturation process called capacitation, which primes the sperm to navigate across the oviduct and fertilize the egg. Sperm capacitation and motility are controlled by 3′,5′-cyclic adenosine monophosphate (cAMP). Here, we show that optogenetics, the control of cellular signaling by genetically encoded light-activated proteins, allows to manipulate cAMP dynamics in sperm flagella and, thereby, sperm capacitation and motility by light. To this end, we used sperm that express the light-activated phosphodiesterase LAPD or the photo-activated adenylate cyclase bPAC. The control of cAMP by LAPD or bPAC combined with pharmacological interventions provides spatiotemporal precision and allows to probe the physiological function of cAMP compartmentalization in mammalian sperm.

## 1. Introduction

The sperm flagellum functions as both sensor and propeller, allowing sperm to steer along physical and chemical cues provided by the female genital tract towards the site of fertilization [1]. To this end, sensory signaling pathways in the flagellum translate the complex physico-chemical code of the oviductal microenvironment into motor responses [2]. The second messenger cyclic AMP (cAMP) plays a key role for sperm motility [3]: Genetic ablation of the predominant source of cAMP in mammalian sperm, the bicarbonate-dependent, soluble adenylate cyclase Adcy10, renders sperm immotile [4,5,6]. Bicarbonate-induced cAMP synthesis by Adcy10 triggers the phosphorylation of flagellar motor proteins by protein kinase A (PKA), increasing the flagellar beat frequency [7,8,9,10]. PKA-dependent protein phosphorylation also stimulates the activity of the tyrosine kinase FER [11]. FER-mediated protein tyrosine phosphorylation is a hallmark of sperm capacitation, a maturation process inside the female genital tract. In vitro, capacitation can be induced by incubation of sperm in medium containing Ca^2+^, bicarbonate, an energy source (e.g., lactate, glucose), and a cholesterol acceptor (e.g., serum albumin) [12]. 

In the last few years, the picture emerged that cAMP signaling in sperm is compartmentalized, i.e., in head versus flagellum, with the latter being further separated into the midpiece, principal piece, and endpiece [3,13,14,15]. To decipher the underlying molecular principles, cAMP signaling needs to be dissected with spatiotemporal precision. Optogenetics and genetically-encoded biosensors have already been proven as powerful tools to manipulate and analyze cAMP signaling in mouse sperm [13,15]. The photo-activated adenylate cyclase bPAC from *Beggiatoa* sp. [16] makes it possible to control cAMP dynamics and sperm motility by light [13], and cAMP dynamics along the flagellum can be monitored with a FRET-based cAMP biosensor [15]. Here, to complement the tool set, we expressed the light-activated phosphodiesterase LAPD [17] in mouse sperm to manipulate cAMP signaling in the flagellum. We demonstrate that the control of cAMP levels by LAPD can be used to study the role of cAMP for sperm capacitation and that the combination of optogenetics and pharmacology is well-suited to study the cAMP-dependent control of sperm motility. 

## 2. Material and Methods

### 2.1. Mouse Sperm

Animal care and experiments were approved by the local authorities (Landesamt für Natur, Umwelt und Verbraucherschutz, North Rhine-Westphalia, LANUV). Mice were killed by cervical dislocation after isoflurane (Curamed Pharma, Karlsruhe-Durlach, Germany) inhalation under normal room light for protein-phosphorylation analysis and under dim green light for sperm imaging. Sperm were isolated by incision of the cauda epididymis followed by a swim-out in modified TYH medium (in mM: 135 NaCl, 4.8 KCl, 2 CaCl_2_, 1.2 KH_2_PO_4_, 1 MgSO_4_, 5.6 glucose, 0.5 sodium pyruvate, 10 lactic acid, 10 HEPES, pH 7.4 adjusted at 37 °C with NaOH). After 15–30 min swim-out at 37 °C, sperm were collected and counted.

### 2.2. Generation of Transgenic Mice

**Figure 1:**
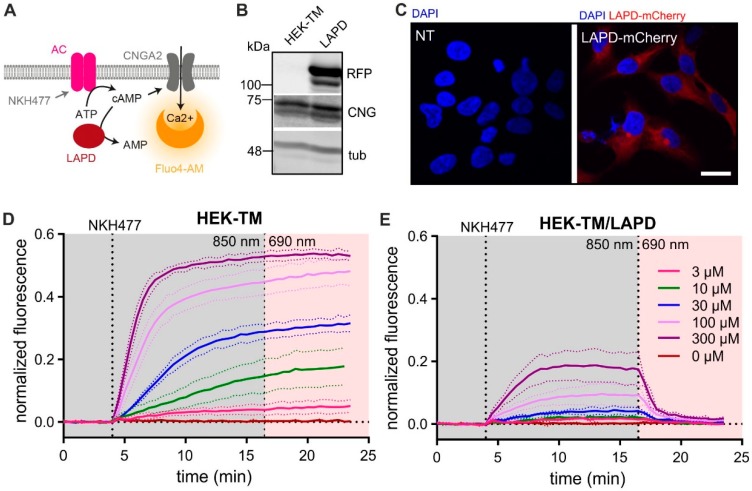
Characterization of LAPD in HEK-TM cells. (**A**) Scheme illustrating the principle of the assay used to measure LAPD activity in HEK cells via cAMP-gating of a Ca^2+^-permeable cyclic nucleotide-gated ion channel (CNGA2-TM) [19]. HEK-TM cells were first stimulated with 100 μM NKH477 to activates transmembrane adenylate cyclases (AC). The ensuing increase in cAMP evokes a Ca^2+^ influx that is detected by a fluorescent Ca^2+^ indicator (Fluo4-AM). Subsequently, cells were illuminated to stimulate LAPD activity. (**B**) Western blot analysis of LAPD-mCherry expression in HEK-TM cells (LAPD). Non-transfected cells (HEK-TM) were used as a control. Immunoblots were stained with RFP, CNGA2, and alpha-tubulin antibodies. (**C**) Immunocytochemical analysis of LAPD-mCherry expression in HEK-TM cells. Non-transfected cells (NT) were used as a control. Cells were stained with DAPI (blue) to label the DNA; scale bar: 10 μm. (**D**,**E**) Measuring LAPD activity. Fluo4-AM-loaded HEK-TM cells were incubated with increasing concentrations of NKH477 (see legend in E) during continuous 850 nm light illumination (grey, 0.5 µW/cm^2^). When reaching a steady-state, light was switched to 690 nm (red, 0.5 µW/cm^2^) to stimulate LAPD activity. To evoke a maximal Ca^2+^ response, cells were stimulated at the end of the experiment with 2 μM ionomycin for normalization (data not shown). Data are shown as mean ± S.D. Panels (D) and (E) show data for non-transfected HEK-TM cells and stable LAPD-mCherry lines, respectively.

The LAPD cDNA sequence [17] was amplified via PCR. A hemagglutinin (HA) tag was fused to the C terminus, an EcoRI restriction site was added to the 5′ end, and a BamHI and XhoI restriction site to the 3′ end by nested PCR. The PCR product was cloned into a pBluescript SK^−^ vector (Agilent Technologies, Santa Clara, CA, USA) using EcoRI and XhoI (pB-LAPD). After sequencing, the LAPD-HA insert was excised and cloned into pPrCExV (kind gift from Bob Brown, Jackson Laboratory) using EcoRI/BamHI to express LAPD under the control of the protamine-1 promotor that is exclusively active in post-meiotic spermatids. Transgenic mice were generated via pronuclear injection at the transgenic facility of the LIMES (University of Bonn, Germany; license number: 84–02.04.2012.A192). Mice were genotyped by PCR using LAPD-specific primers (#1: GGACTGATCCTGAGATTTGAGGGC, #2: CAGCCACAGGCTGTATCCCATCATG; see also Figure 1A). Mice used in this study were 2–5 months of age. 

### 2.3. LAPD Activity Assay

For PDE activity assays in eukaryotic cells, cells were seeded on a PLL-coated 96-well plate at 3 × 10^4^ cells per well and incubated over night at 37 °C and 5% (*v/v*) CO_2_ in darkness. All following steps were conducted under dim green light. Medium was removed, and cells were washed with 50 μL ES buffer (120 mM NaCl, 5 mM KCl, 2 mM CaCl_2_, 2 mM MgCl_2_, 10 mM glucose, 10 mM HEPES pH 7.4). Cells were loaded with 2 μM Fluo4-AM (stock in DMSO/pluronic) and 3 mM probenecid in 50 µL ES for 30 min at 37 °C. Afterwards, the buffer was replaced with 90 μL ES containing 3 mM probenecid, and cells were incubated for 30 min at 29 °C inside the fluorescence plate-reader (FLUOstar omega, BMG Labtech). Fluorescence was measured at 29 °C with the 485 ± 6 excitation and 530 ± 15 nm emission wavelengths. After 2 min, NKH477 (Sigma-Aldrich, MI, USA) in ES buffer was added (final concentration: 100 µM), and measurements continued for 15 min. In the controls, only ES buffer was added. During the measurement, the plate was illuminated with an 850-nm LED (0.5 μW/cm^2^) mounted inside the reader until the illumination was switched to a 690-nm LED (0.5 μW/cm^2^) to activate LAPD. After another 6 min, ionomycin was added (final concentration: 2 µM), and fluorescence was recorded until saturation of the signal amplitude.

### 2.4. Western Blot Analysis

Protein lysates were obtained by homogenizing cells or tissue in lysis buffer (10 mM Tris/HCl, pH 7.6, 140 mM NaCl, 1 mM EDTA, 1% Triton X-100, mPIC protease inhibitor cocktail 1:500, Sigma-Aldrich, MI, USA). Samples were incubated for 30 min on ice and centrifuged at 10,000× *g* for 5 min at 4 °C. Prior to separation by SDS-PAGE, samples were mixed with 4 × SDS loading-buffer (200 mM Tris/HCl, pH 6.8, 8% SDS (*w/v*), 4% β-mercaptoethanol (*v/v*), 50% glycerol, 0.04% bromophenol blue) and heated for 5 min at 95 °C. Sperm samples used for SDS-PAGE were washed with 1 mL PBS and sedimented by centrifugation at 5000× *g* for 5 min. 1–2 × 10^6^ cells were resuspended in 2 × SDS loading buffer and heated for 5 min at 95 °C. For Western blot analysis, proteins were transferred onto PVDF membranes (Merck Millipore, Billerica, USA), probed with antibodies, and analyzed using the Odyssey Imaging System (LI-COR Biosciences, Bad Homburg, Germany). To analyze protein tyrosine phosphorylation, 1.5–3 × 10^6^ sperm were incubated in 1.5 mL of capacitation buffer containing TYH buffer plus 25 mM NaHCO_3_ and 3 mg/mL BSA for 0, 60, or 90 min at 37 °C under white light. Sperm were centrifuged at 10,000× *g* for 1 min and frozen in liquid nitrogen. Prior to SDS-PAGE and Western blot analysis, samples were washed in 1 mL PBS, resuspended in 2× SDS loading buffer, and heated for 5 min at 95 °C. The pY and alpha-tubulin band intensities of WT and LAPD samples after 60 min capacitation were quantified using ImageJ (1.52i, U.S. National Institutes of Health, Bethesda, MD, USA). The pY band intensities of WT and LAPD sperm were normalized to the intensity of the alpha-tubulin loading control and plotted as a ratio of LAPD/WT.

The following antibodies were used for Western blot analysis: (a) primary antibodies: HA 3F10 antibody (1:5000, rat monoclonal; Roche, Basel, Switzerland), alpha-tubulin antibody (1:5000, mouse monoclonal, clone B–5–1–2, T5168; Sigma-Aldrich, MI, USA), phospho-tyrosine 4G10 antibody (1:1000, mouse monoclonal, 05–321; Merck, NJ, USA); (b) secondary antibodies: IRDye680 and IRDye800 antibodies (LI-COR, 1:20,000).

### 2.5. Immunohistochemistry and Immunocytochemistry

Testes were fixed in 4% paraformaldehyde/PBS overnight, cryo-protected first in 10% and then in 30% sucrose, and embedded in Tissue Tek (Sakura Finetek). To block unspecific binding sites, cryosections (16 µm) were incubated for 1 h with blocking buffer (0.5% Triton X-100 and 5% ChemiBLOCKER (Merck Millipore, Billerica, MA, USA) in PBS, pH 7.4). Primary antibodies were diluted in blocking buffer and incubated for 2 h. Fluorescent secondary antibodies were diluted in blocking buffer containing 0.5 mg/mL DAPI (Life Technologies) and pictures were taken on a confocal microscope (FV1000; Olympus). For analyzing protein tyrosine phosphorylation, band intensities of the whole lane were determined with ImageJ (1.52i, U.S. National Institutes of Health, Bethesda, MD, USA).

2 × 10^5^ sperm were plated and allowed to dry on glass slides. Dried sperm were rinsed twice with PBS for 15 min and heated for 20 min at 99 °C in 10 mM sodium citrate solution (pH 6, with 0.05% Tween-20) to retrieve the antigen. After cooling the slides in PBS, sperm were fixed in 4% paraformaldehyde for 15 min and washed thrice in PBS containing 0.1% Triton X-100 (washing solution). Unspecific binding sites were blocked by incubating slides for 1 h in blocking solution (PBS containing 1% BSA and 0.1% Triton X-100). Primary antibodies diluted in blocking solution were incubated overnight. Afterwards, sperm were washed thrice in washing solution and incubated overnight, with fluorescently labelled secondary antibodies diluted in blocking solution. After washing, sperm were incubated for 45 min with 0.5 mg/mL DAPI diluted in PBS and mounted (Aqua Poly/Mount, Polysciences, Hirschberg an der Bergstrasse, Germany). Image recording was performed as mentioned above. 

The following antibodies were used: (a) primary antibodies: HA 3F10 antibody (1:1000, rat monoclonal; Roche, Basel, Switzerland), tubulin, PDE2A antibody (1:250, rabbit polyclonal, PD2A–101AP; Fabgennix, Texas, USA); beta-tubulin-cy3 antibody (1:500, mouse monoclonal, clone TUB 2.1, C4585; Sigma-Aldrich, Missouri, USA); (b) secondary antibodies: donkey anti-rat CY3 (1:500, Dianova, Hamburg, Germany), anti-rabbit 488 (1:500, Dianova, Hamburg, Germany).

### 2.6. Determination of Total cAMP Content

Prm1-LAPD (1 × 10^6^) sperm were illuminated with 690 nm light for 5 min. Afterwards, sperm were quenched with 0.5 M HClO_4_, vortexed, frozen in liquid N_2_, thawed, and neutralized by addition of K_3_PO_4_ (0.24 M final concentration). The salt precipitate and cell debris were sedimented by centrifugation (15 min, 20,000× *g*, 4 °C). The cAMP content in the supernatant was determined by a competitive immunoassay (CatchPoint cAMP Fluorescent Assay Kit, Molecular Devices, San Jose, USA) including an acetylation step for higher sensitivity. Calibration curves were obtained by serial dilutions of cAMP standards. 96-well plates were analyzed by using a microplate reader (FLUOstar Omega; BMGLabtech, Ortenberg, Germany).

### 2.7. Imaging

Dark-field microscopy was performed using an inverted microscope (IX71; Olympus, Hamburg, Germany) equipped with a dark-field condenser and a high-speed camera (ORCA-Flash4.0 V3, C13220–20 CU, Hamamatsu, Hamamatsu City, Japan). Imaging of Prm1-bPAC sperm was performed as described previously [13]. Image sequences of wild-type and Prm1-LAPD sperm were recorded with 200 frames per second (fps) using a 10× objective (NA 0.4, UPlanFL; Olympus, Hamburg, Germany) with an additional 1.6× magnifying lens (Olympus, Hamburg, Germany) that was inserted into the light path (final magnification: 16×). For imaging, sperm purified by swim-up were added to an ibidi chamber with a depth of about 400 µm (M-SLIDE VI, Elvesys, Paris, France). The temperature of the microscope incubator (Life Imaging Services, Basel, Switzerland) was adjusted to 37 °C for imaging. To image tethered sperm, cells were perfused into the chamber using a syringe pump and the flagellar beat was recorded under illumination at 850 nm. TYH buffer containing 25 mM NaHCO_3_ was perfused into the chamber and after 30 s, the flagellar beat of the sperm in buffer with high bicarbonate was recorded under 850 nm illumination. Sperm were then illuminated with 660 nm light and the beat frequency was recorded after 1 min of illumination. Imaging was performed under green room light. Imaging of Prm1-bPAC sperm has been described before [13]. The beat frequency was determined using SpermQ [18]; the details are as follows. At each flagellar position, the time-course of the parameter curvature angle was converted into a frequency spectrum by Fast-Fourier-Transformation (integrated in SpermQ). The frequency of the highest (f1) and second highest (f2) peak in the power spectrum was reported [18] and merged into a local beat frequency value as (f1 + f2)/2. The flagellar beat frequency of each sperm was determined as the average local beat frequency of all flagellar positions between 15 and 60 µm. For data presented in Figure 4D–F, every frame represents one beat cycle and all images were recorded at 200 Hz. Because the beat frequency was different under different conditions, different numbers of frames were used to create a one-beat-cycle overlay. The number of frames was adjusted to the number of frames corresponding to one beat cycle (= recording frequency/beat frequency).

### 2.8. Image Analysis

All image processing and analysis was performed in ImageJ (1.52i, U.S. National Institutes of Health, Bethesda, MD, USA). Image Sequences of sperm were cropped to single-sperm images, which were further processed by a Gaussian Blur (sigma: 0.5) and background-removed using the ImageJ function Subtract Background (radius: 5 px, corresponding to 3.25 µm). After background correction, image sequences were subjected to SpermQ analysis (settings: Table 1) [18]. The local beat asymmetry was determined using SpermQ_Evaluator; the local beat asymmetry at a given point of the sperm flagellum represents the absolute of the average of all curvature angle values over time at the given point.

### 2.9. Software Availability

SpermQ is accessible upon request [18]. SpermQ Evaluator is publicly available at https://github.com/IIIImaging/SpermQ_Evaluator.

## 3. Results

### 3.1. Characterization of Heterologously Expressed LAPD

First, we scrutinized whether LAPD can be functionally expressed in a mammalian system using HEK-293 cells. To this end, we stably co-expressed a LAPD-mCherry fusion protein with a cyclic nucleotide-gated (CNGA2-TM) ion channel (HEK-TM). The Ca^2+^-permeable CNG channel is gated by cAMP and, thereby, translates changes of the intracellular cAMP concentration into changes of the intracellular Ca^2+^ concentration, which can be monitored with a fluorescent Ca^2+^ indicator [19]. In HEK-TM cells, both in the absence (control) and presence of LAPD, activation of transmembrane adenylate cyclases by the water-soluble forskolin analog NKH477 evoked a sustained Ca^2+^ increase, whose amplitude rose in a dose-dependent fashion (Figure 1D,E). The cells were continuously illuminated with 850 nm light to keep LAPD in a low-activity state [17]. Yet, the expression of LAPD attenuated the amplitude of the NKH477 response, indicating that a basal “dark” activity of LAPD counteracted cAMP synthesis evoked by NKH477 (Figure 1E). When the NKH477 response reached a steady-state, LAPD was activated by 680 nm light. In turn, in LAPD-expressing, but not in control cells, Ca^2+^ levels decreased due to light-induced cAMP hydrolysis (Figure 1D,E). After light-induced activation of LAPD, the Ca^2+^ and, thus, cAMP concentration resumed to levels prevailing before NKH477 stimulation. The slope of the Ca^2+^ decrease was correlated with the NKH477 concentration, i.e., with increasing “resting” cAMP levels, the LAPD-mediated cAMP decrease was faster (Figure 1E). In summary, our results demonstrate that LAPD is functional in mammalian cells, which allowed us to design similar experiments in mouse sperm.

### 3.2. Generation of Transgenic Mice

**Figure 2:**
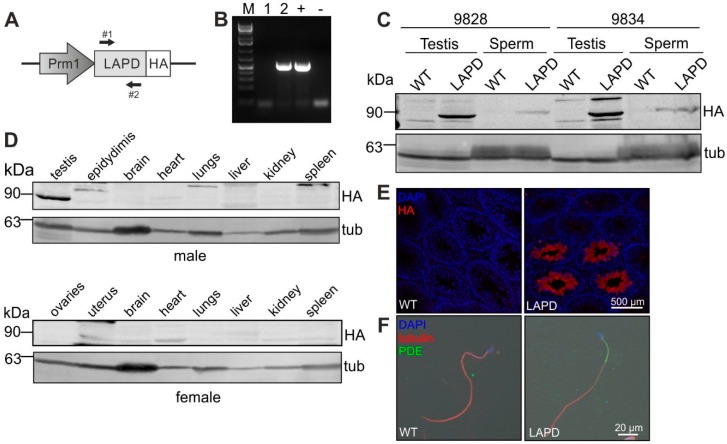
Design and characterization of Prm1-LAPD mice. (**A**) Scheme of the Prm1-LAPD targeting vector. Expression of hemagglutinin (HA)-tagged LAPD is driven by the Protamine 1 promoter (Prm1); arrows indicate the position of genotyping primers. (**B**) Genotyping by PCR. In Prm1-LAPD mice, a 514 bp fragment is amplified. (1) Wild-type mouse, (2) Prm1-LAPD mouse; the targeting vector served as a positive control (+) and H_2_O as negative control (−). (**C**) Western blot analysis of LAPD-HA expression in testis and sperm of wild-type (WT) and Prm1-LAPD mice (LAPD) from two different founder lines. Immunoblots were stained with HA and alpha-tubulin antibodies. (**D**) Western blot analysis of LAPD-HA expression in various tissues from male and female Prm1-LAPD mice. Immunoblots were stained with HA and alpha-tubulin antibodies. (**E**) Cryo-sections from wild-type (WT) and Prm1-LAPD (LAPD) testes labeled with a HA antibody (red) and DAPI (blue). (**F**) Wild-type (WT) and Prm1-LAPD (LAPD) sperm labeled with PDE2A (green) and alpha-tubulin antibodies (red), and DAPI (blue). Scale bars are indicated.

To manipulate cAMP levels in vivo in mammalian sperm, we generated transgenic mice expressing LAPD under the control of the protamine 1 promoter (Prm1, Figure 2A). Genomic insertion of the transgene was confirmed by PCR (Figure 2B). We analyzed two founders (9828 and 9834), none of which showed any gross phenotype. Moreover, the fecundity of transgenic males was similar to that of wild-type males. In Prm1-LAPD mice, LAPD was exclusively expressed in sperm, predominantly in the midpiece of the flagellum (Figure 2C–F).

### 3.3. Characterization of LAPD Function in Mouse Sperm

**Figure 3:**
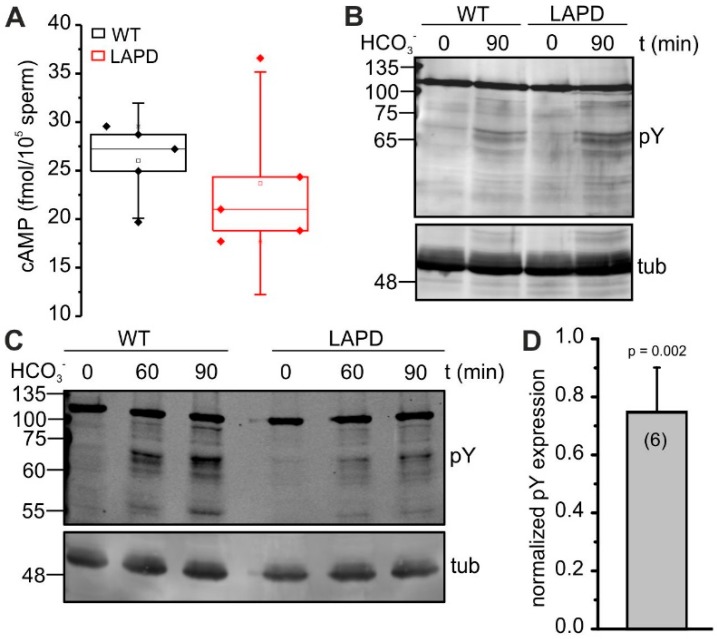
Analysis of LAPD function in mouse sperm. (**A**) Total cAMP content in wild-type (WT) and Prm1-LAPD sperm after stimulation with 690 nm light for 5 min. Data are shown as box plots, containing individual data points and mean ± SD (Per 25/75). (**B**) Western blot analysis of protein tyrosine phosphorylation. Tyrosine phosphorylation after stimulation with 25 mM NaHCO_3_ for 0 and 90 min in the dark in wild-type (WT) and Prm1-LAPD (LAPD) sperm was detected with a phospho-tyrosine antibody (pY). Alpha-tubulin was used as loading control. (**C**) See (B) after continuous white light stimulation for 0, 60, and 90 min. (**D**) Quantification of protein tyrosine phosphorylation in Prm1-LAPD sperm, relative to wild-type sperm (set to 1), after 90 min bicarbonate incubation under 660 nm illumination. Data were normalized to the WT. Data are shown as mean + SD, n number is indicated.

We investigated whether light-dependent activation of LAPD affects cAMP levels in sperm. To this end, we first determined via ELISA the total cAMP content in wild-type and Prm1-LAPD sperm after illumination at 690 nm to activate LAPD. In Prm1-LAPD sperm, the total cAMP content was seemingly, but not statistically significantly, reduced (*p* = 0.5, Figure 3A). However, the basal concentration of cAMP in mouse sperm is low [15], which might hamper the detection of a LAPD-mediated decrease of total cAMP content. Thus, we tested whether light-activation of LAPD is able to counteract the bicarbonate-induced increase in cAMP. To this end, we analyzed the FER-mediated increase in protein tyrosine phosphorylation, which is triggered by PKA and a hallmark of capacitation [20]. We investigated the time course of bicarbonate-induced tyrosine phosphorylation in wild-type and Prm1-LAPD sperm in the dark and after light stimulation. In the dark, tyrosine phosphorylation increased in both, wild-type and Prm1-LAPD sperm. The intensity of the band pattern was slightly more enhanced in Prm1-LAPD sperm compared to wild-type sperm, which could be attributed to a compensatory response due to dark activity of LAPD (Figure 3B). After light stimulation, tyrosine phosphorylation was, however, strongly attenuated in Prm1-LAPD sperm (Figure 3C–D), indicating that light-dependent activation of LAPD can counteract bicarbonate-induced cAMP synthesis. 

### 3.4. Controlling Sperm Motility by Optogenetics

In addition to protein tyrosine phosphorylation, the flagellar beat frequency is also controlled by cAMP [7,8,13]: in wild-type sperm, bicarbonate-induced cAMP synthesis increased the flagellar beat frequency from 11 ± 4 Hz to 22 ± 6 Hz (Supplementary Movie 1, *n* = 22). Of note, inhibition of cAMP hydrolysis by PDEs using IBMX did not significantly change the beat frequency of wild-type sperm (control: 10 ± 4 Hz vs. IBMX: 10 ± 3 Hz, *n* = 12), indicating that the basal cAMP-synthesis rate is rather low in mouse sperm. The beat frequency of Prm1-LAPD sperm was similar to wild-type sperm, both at 850 nm and 660 nm illumination to suppress and stimulate LAPD activity, respectively (Supplementary Movie 2; 10 ± 4 Hz, *n* = 30 vs. 10 ± 3 Hz, *n* = 11). However, bicarbonate was seemingly more efficacious to increase the beat frequency in Prm1-LAPD sperm than in wild-type sperm: Under 850 nm light, the flagellar beat frequency of Prm1-LAPD increased from 10 ± 3 Hz to 27 ± 12 Hz (Supplementary Movie 3, *n* = 22), whereas in wild-type sperm, the frequency increased from 11 ± 4 to 22 ± 6 Hz (*n* = 25, *p* = 0.06). Thus, for unknown reasons, the expression of LAPD renders the motility apparatus more sensitive to an increase in the intracellular cAMP concentration. In the presence of bicarbonate, however, light-induced activation of LAPD did decrease the flagellar beat frequency (LAPD: 24 ± 9 Hz vs. WT: 22 ± 12 Hz, *n* = 22), indicating that cAMP hydrolysis by LAPD, due to its high Km value, is not sufficient to counteract the bicarbonate action on beat frequency. 

**Figure 4:**
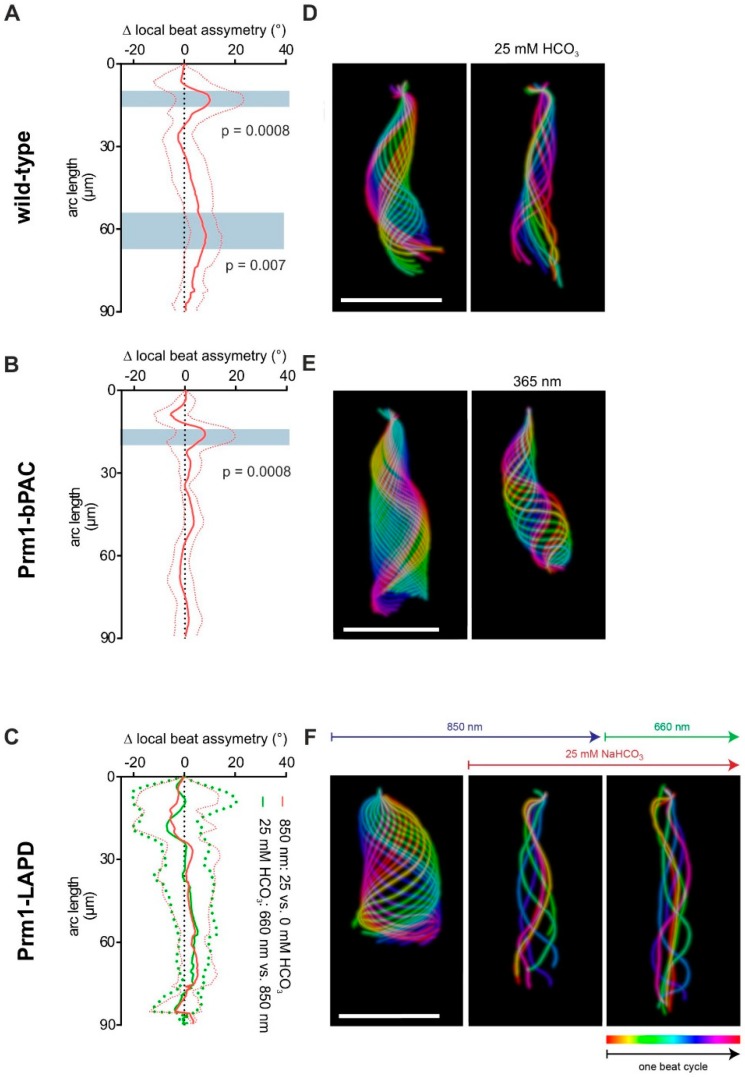
Using optogenetics to control sperm motility. (**A**) Local beat asymmetry, determined at each flagellar position by the absolute of the time-averaged curvature angle. Wild-type sperm (WT) were analyzed before and 1 min after stimulation with 25 mM NaHCO_3_ (850 nm). Plotted is the difference before and after stimulation as mean (dark red line) ± S.D. (light red line). (**B**) Local beat asymmetry Prm1-bPAC sperm before and after 10 s exposure to 365 nm light. Plotted is the difference after compared to before stimulation as mean (dark red line) ± S.D. (light red line). (**C**) Local beat asymmetry for Prm1-LAPD sperm at 850 nm illumination before and after stimulation with 25 mM NaHCO_3_, and 1 min after switching to 660 nm illumination in the presence of 25 mM NaHCO_3_. Plotted is the difference after compared to before stimulation with 25 mM NaHCO_3_ (red), and after compared to before switching to 660 nm (green). Data are shown as mean ± S.D. (dotted line) and have been determined using SpermQ and SpermQ_Evaluator; *p*-values were determined using a Two-Way ANOVA and corrected for multiple testing (Turkey’s range test); regions with significant test results (*p* < 0.05) are highlighted in grey and indicated *p*-values refer to the lowest *p*-value within the highlighted range. (**D**–**F**) Projection of the flagellar waveform for one beat cycle. Each frame is color-coded. (D) for (A); (E) for (B); (F) for (C). Scale bar: 50 μm.

The cAMP dynamics in the sperm flagellum also regulate the flagellar waveform [8]. Therefore, using SpermQ [18], we investigated cAMP-induced changes in beat symmetry in wild-type, Prm1-LAPD, and Prm1-bPAC sperm, expressing bPAC in the flagellum [13]. As a parameter for the local asymmetry of the flagellar beat, at each flagellar point, SpermQ determines the absolute of the time-average of all curvature angle values. When the beat at a given position along the flagellum is perfectly symmetric, the time-average of all curvature angles is zero, while it increases when the beat becomes asymmetric. In wild-type and Prm1-bPAC sperm, bicarbonate- and light-induced cAMP synthesis, respectively, increased the local beat asymmetry in the first 30 μm of the flagellum (Figure 4A,B). Bicarbonate-stimulation of wild-type, but not light-stimulation of Prm1-bPAC sperm increased the local beat asymmetry also in the distal part of the flagellum (at ~60 μm; Figure 4A). In Prm1-LAPD sperm, bicarbonate did not affect the beat asymmetry at any point along the flagellum, neither under control conditions (850 nm) nor under 660 nm illumination (Figure 4C). The action of the bicarbonate- and light-induced increase of cAMP are also evident in the projection of the flagellar waveform for one beat cycle for each condition and mouse line (Figure 4D–F). Thus, manipulating cAMP dynamics using either pharmacology or optogenetics results in a distinct response in the flagellar waveform. This will make it possible to design experiments to decipher the compartmentalization of cAMP signaling along the flagellum.

## 4. Discussion

Mammalian sperm function relies on cAMP signaling pathways. Here, we establish and apply an optogenetic tool box to manipulate cAMP signaling in mouse sperm by light. 

In mammalian sperm, cAMP-signaling components are not uniformly distributed; PKA and Adcy10 are exclusively localized to the flagellum [5,10,21] and cAMP signaling is compartmentalized along the flagellum. Live-cell cAMP imaging using a FRET-based cAMP biosensor revealed that the kinetics of the bicarbonate-induced cAMP changes are faster in the midpiece compared to the principal piece [15]. In fact, this compartmentalization might be the basis for the differential cAMP response of the flagellar waveform in the distal and proximal part of the flagellum upon bicarbonate stimulation. The local beat asymmetry in the midpiece displays a more profound increase than in the principal piece (see Figure 4A). The domain architecture might be due to a distinct flagellar distribution of PDEs [22]: at least two PDE isoforms and splice variants, PDE4 (cAMP-specific) and PDE1 (Ca^2+^/calmodulin-dependent), control sperm capacitation and motility [23,24,25]. An enhanced PDE activity in certain flagellar compartments might restrict free diffusion of cAMP, creating cAMP microdomains. In the presence of LAPD, the bicarbonate response is abolished altogether, indicating that the expression of LAPD interferes with cAMP signaling and its compartmentalization. Light-stimulation of bPAC or LAPD activity in the whole flagellum most likely overrides the endogenous cAMP machinery and, thereby, disables the microdomain-dependent control of the flagellar waveform. Thus, illuminating the sperm flagellum with spatial precision might make it possible to analyze the function and molecular underpinnings of cAMP compartments on sperm motility in more detail. 

Understanding how cAMP microdomains along the flagellum control sperm motility is particularly important to unravel how sperm navigate. Various chemical and physical cues guide sperm to the egg. In mammals, three guidance mechanisms have been proposed: chemotaxis, rheotaxis, and thermotaxis [1,26]. However, the molecular mechanisms underlying mammalian sperm motility are ill-defined. Whether and how cAMP signaling is involved in any of these guidance mechanisms is unknown. It has been proposed that progesterone-dependent chemotaxis in human sperm is controlled by a cAMP-dependent signaling pathway that involves activation of PKA [27]. However, this concept could not be confirmed by others [28]. Furthermore, G-protein coupled signaling pathways that act via cAMP have also been proposed to govern thermotaxis in mammalian sperm [29]. Thus, to gain further insight in to sperm physiology and the fertilization process, the role of cAMP-signaling pathways has to be elucidated and the application of optogenetic tools will be a great asset to this endeavor. 

Understanding the molecular principles underlying sperm motility also goes beyond basic research. Infertility affects approximately 15% of all couples wanting to conceive. In 30–40% of cases, the etiology of male infertility remains unknown and is called idiopathic male infertility. The success rates of assisted reproductive technologies (ART) highly depends on understanding the molecular mechanisms underlying spermatogenesis and sperm function. Thereby, the diagnosis can be improved and a more personalized treatment can be developed. Men treated with PDE5 inhibitors, e.g., vardenafil, displayed increased cAMP levels in sperm and improved sperm motility, suggesting that PDE5 inhibitors may improve the outcome of ART programs [30]. Furthermore, cAMP-dependent signaling is crucial for sperm development. The cAMP-dependent transcription factor CREM regulates the expression of genes that control spermiogenesis, the structuring of the mature sperm cell. In normospermic men, a switch from expression of repressors to activators occurs, which is regulated by CREM-dependent gene expression. Patients that show a maturation arrest during sperm development only express repressors, but no activators, demonstrating the importance of cAMP-dependent gene expression during sperm development [31]. Thus, understanding the molecular details of cAMP signaling in sperm is clinically relevant and might improve diagnoses and treatment of male infertility. 

## Figures and Tables

**Table 1:** SpermQ settings.

Parameter	Setting
Thresholding Method	Triangle
Gauss Sigma	1.0
Repeat gauss fit after binarization	false
Blur only inside ROI selection	false
Upscaling of point list (fold)	3
Add head center-of-mass as first point	false
Unify start points	false
Filter points by gauss fits	false
Maximum vector length (points)	14
Normal radius for gauss fit (µm)	5.0
Exclude head from correction or deletion	true
Smooth normal for XY gauss fit	true
Accepted xy distance of points for fit-width-smoothing (µm)	9.6
# (+/−)-consecutive points for xy- and fit-width-smoothing	15
Distance of point to first point to form the reference vector (µm)	10.0
Curvature: reference point distance	10.0
FFT: Grouped consecutive time-steps	400
FFT: Do not analyze (frequency results of flagellar parameters for the) initial … µm from head	0.0
Head rotation matrix radius	10

## Supplementary Materials

The following are available online at http://www.mdpi.com/2073-4409/8/7/648/s1. The manuscript contains Supplementary Movies 1–3.

## References

[1] Wachten D., Jikeli J.F., Kaupp U.B. (2017). Sperm Sensory Signaling. Cold Spring Harb. Perspect. Biol..

[2] Alvarez L., Friedrich B.M., Gompper G., Kaupp U.B. (2014). The computational sperm cell. Trends Cell. Biol..

[3] Balbach M., Beckert V., Hansen J.N., Wachten D. (2018). Shedding light on the role of cAMP in mammalian sperm physiology. Mol. Cell. Endocrinol..

[4] Esposito G., Jaiswal B.S., Xie F., Krajnc-Franken M.A.M., Robben T.J.A.A., Strik A.M., Kuil C., Philipsen R.L.A., Van Duin M., Conti M. (2004). Mice deficient for soluble adenylyl cyclase are infertile because of a severe sperm-motility defect. Proc. Natl. Acad. Sci. USA.

[5] Hess K.C., Jones B.H., Marquez B., Chen Y., Ord T.S., Kamenetsky M., Miyamoto C., Zippin J.H., Kopf G.S., Suarez S.S. (2005). The “Soluble” Adenylyl Cyclase in Sperm Mediates Multiple Signaling Events Required for Fertilization. Dev. Cell.

[6] Xie F., Garcia M.A., Carlson A.E., Schuh S.M., Babcock D.F., Jaiswal B.S., Gossen J.A., Esposito G., Van Duin M., Conti M. (2006). Soluble adenylyl cyclase (sAC) is indispensable for sperm function and fertilization. Dev. Boil..

[7] Carlson A.E., Hille B., Babcock D.F. (2007). External Ca^2+^ acts upstream of adenylyl cyclase SACY in the bicarbonate signaled activation of sperm motility. Dev. Biol..

[8] Wennemuth G., Carlson A.E., Harper A.J., Babcock D.F. (2003). Bicarbonate actions on flagellar and Ca^2+^-channel responses: Initial events in sperm activation. Development.

[9] Lackey B.R., Gray S.L. (2015). Identification of kinases, phosphatases, and phosphorylation sites in human and porcine spermatozoa. Syst. Boil. Reprod. Med..

[10] Nolan M.A., Babcock D.F., Wennemuth G., Brown W., Burton K.A., McKnight G.S. (2004). Sperm-specific protein kinase A catalytic subunit Ca^2+^ orchestrates cAMP signaling for male fertility. Proc. Natl. Acad. Sci. USA.

[11] Alvau A., Battistone M.A., Gervasi M.G., Navarrete F.A., Xu X., Sánchez-Cárdenas C., De La Vega-Beltran J.L., Da Ros V.G., Greer P.A., Darszon A. (2016). The tyrosine kinase FER is responsible for the capacitation-associated increase in tyrosine phosphorylation in murine sperm. Develpoment.

[12] Suarez S.S. (2008). Control of hyperactivation in sperm. Hum. Reprod. Updat..

[13] Jansen V., Álvarez L., Balbach M., Strünker T., Hegemann P., Kaupp U.B., Wachten D., Clapham D.E. (2015). Controlling fertilization and cAMP signaling in sperm by optogenetics. ELife.

[14] Jansen V., Jikeli J.F., Wachten D. (2017). How to control cyclic nucleotide signaling by light. Curr. Opin. Biotechnol..

[15] Mukherjee S., Jansen V., Jikeli J.F., Hamzeh H., Alvarez L., Dombrowski M., Balbach M., Strünker T., Seifert R., Kaupp U.B. (2016). A novel biosensor to study cAMP dynamics in cilia and flagella. ELife.

[16] Stierl M., Stumpf P., Udwari D., Gueta R., Hagedorn R., Losi A., Gärtner W., Petereit L., Efetova M., Schwarzel M. (2011). Light modulation of cellular cAMP by a small bacterial photoactivated adenylyl cyclase, bPAC, of the soil bacterium Beggiatoa. J. Biol. Chem..

[17] Gasser C., Taiber S., Hegemann P., Ryu S., Wunder F., Möglich A., Yeh C.-M., Wittig C.H. (2014). Engineering of a red-light-activated human cAMP/cGMP-specific phosphodiesterase. Proc. Natl. Acad. Sci. USA.

[18] Hansen J.N., Rassmann S., Jikeli J.F., Wachten D. (2018). SpermQ–A Simple Analysis Software to Comprehensively Study Flagellar Beating and Sperm Steering. Cells.

[19] Wachten S., Schlenstedt J., Gauss R., Baumann A. (2006). Molecular identification and functional characterization of an adenylyl cyclase from the honeybee. J. Neurochem..

[20] Morgan D.J., Weisenhaus M., Shum S., Su T., Zheng R., Zhang C., Shokat K.M., Hille B., Babcock D.F., McKnight G.S. (2008). Tissue-specific PKA inhibition using a chemical genetic approach and its application to studies on sperm capacitation. Proc. Natl. Acad. Sci. USA.

[21] Wertheimer E., Krapf D., De La Vega-Beltran J.L., Sánchez-Cárdenas C., Navarrete F., Haddad D., Escoffier J., Salicioni A.M., Levin L.R., Buck J. (2013). Compartmentalization of Distinct cAMP Signaling Pathways in Mammalian Sperm*. J. Boil. Chem..

[22] Bajpai M., Fiedler S.E., Huang Z., Vijayaraghavan S., Olson G.E., Livera G., Conti M., Carr D.W. (2006). AKAP3 selectively binds PDE4A isoforms in bovine spermatozoa. Biol. Reprod..

[23] Fisch J.D., Behr B., Conti M. (1998). Enhancement of motility and acrosome reaction in human spermatozoa: Differential activation by type-specific phosphodiesterase inhibitors. Hum. Reprod..

[24] Leclerc P. (1995). Mouse sperm adenylyl cyclase: General properties and regulation by the zona pellucida. Boil. Reprod..

[25] Visconti P.E., Moore G.D., Bailey J.L., Leclerc P., Connors S.A., Pan D., Olds-Clarke P., Kopf G.S. (1995). Capacitation of mouse spermatozoa. II. Protein tyrosine phosphorylation and capacitation are regulated by a cAMP-dependent pathway. Develpoment.

[26] Hoang H.D., Miller M.A. (2017). Sperm Navigation Mechanisms in the Female Reproductive Tract. Results Probl Cell. Differ..

[27] Teves M.E., Guidobaldi H.A., Uñates D.R., Sánchez R., Miska W., Publicover S.J., Garcia A.A.M., Giojalas L.C. (2009). Molecular Mechanism for Human Sperm Chemotaxis Mediated by Progesterone. PLoS ONE.

[28] Brenker C., Goodwin N., Weyand I., Kashikar N.D., Naruse M., Krähling M., Müller A., Kaupp U.B., Strünker T. (2012). The CatSper channel: A polymodal chemosensor in human sperm. EMBO J..

[29] Pérez-Cerezales S., Boryshpolets S., Afanzar O., Brandis A., Nevo R., Kiss V., Eisenbach M. (2015). Involvement of opsins in mammalian sperm thermotaxis. Sci. Rep..

[30] Dimitriadis F., Giannakis D., Pardalidis N., Zikopoulos K., Paraskevaidis E., Giotitsas N., Kalaboki V., Tsounapi P., Baltogiannis D., Georgiou I. (2008). Effects of phosphodiesterase 5 inhibitors on sperm parameters and fertilizing capacity. Asian J. Androl..

[31] Peri A., Serio M. (2000). The CREM system in human spermatogenesis. J. Endocrinol. Investig..

